# Case report: Myocarditis following COVID-19 protein subunit vaccination

**DOI:** 10.3389/fcvm.2022.970045

**Published:** 2022-09-07

**Authors:** Yi-ming Dong, Xia Liu, Chen-teng Yang, Qian Qi, Wei-bo Shi, Ying-min Li, Min Zuo, Song-jun Wang, Hai-tao Bi, Ru-fei Ma, Guo-zhong Zhang, Bin Cong

**Affiliations:** Hebei Key Laboratory of Forensic Medicine, Collaborative Innovation Center of Forensic Medical Molecular Identification, Department of Forensic Medicine, Hebei Medical University, Shijiazhuang, China

**Keywords:** adverse event, myocarditis, COVID-19, protein subunit vaccine, autopsy

## Abstract

We report findings in a 34-year-old female patient who presented with fulminant myocarditis 8 days after receiving the first dose of the ZF2001 RBD-subunit vaccine against coronavirus disease 2019 (COVID-19). Autopsy showed severe interstitial myocarditis, including multiple patchy infiltrations of lymphocytes and monocytes in the myocardium of the left and right ventricular walls associated with myocyte degeneration and necrosis. This report highlights the details of clinical presentations and autopsy findings of myocarditis after ZF2001 (RBD-subunit vaccine) vaccination. The correlation between vaccination and death due to myocarditis is discussed.

## Introduction

Based on the rapid spread of coronavirus disease 2019 (COVID-19), there is an urgent need for effective vaccines to provide global immunity. Therefore, the world has been racing to develop safe and effective COVID-19 vaccines. Currently, there are six types of COVID-19 vaccines, such as inactivated vaccine, viral vector-based vaccine, subunit vaccine, live-attenuated vaccine, DNA vaccine, and mRNA vaccine (1).

Extensive clinical data suggest a strong association between COVID-19 mRNA vaccination with and myocarditis or myopericarditis (2, 3). There have been several reports of cases of myocarditis and pericarditis following COVID-19 mRNA vaccination, mainly in young males (4–6). However, myocarditis and pericarditis after COVID-19 recombinant protein vaccination has rarely been reported. This report describes a case of myocarditis following ZF2001 (Anhui Zhifei Longcom/Chinese Academy of Sciences) vaccination in a 36-year-old female patient.

## Case presentation

A 36-year-old woman received her first dose of the COVID-19 vaccine (ZF2001) in April 2021. She developed nausea, vomiting, and diarrhea on the 3rd day after vaccination and fever on the 5th day, with the highest temperature of 39.8°C accompanied by chills, dizziness, and nausea. On the 8th day, she visited a local doctor due to vomiting and fever. Her nasopharyngeal swab test for the new coronavirus nucleic acid was negative for the new coronavirus *ORF1ab* gene and the new coronavirus *N* gene. Cardiopulmonary computerized tomography (CT) showed no abnormalities. Abdomen CT showed fatty liver. She was given levofloxacin anti-infective treatment for 3 days, but her fever was persistent. On the 10th day after vaccination, blood chemistry tests showed an elevated myocardial enzyme (peak creatin-kinase 404 U/L, creatin-kinase MB 30 U/L, and lactate dehydrogenase 228 U/L), increased neutrophil ratio (79.1%), increased erythrocyte sedimentation rate (ESR) (70 mm/h), increased fibrinogen content (5.62 g/L), increased activated partial thromboplastin time (36.0 s). Electrocardiogram (ECG) showed bigamy and right bundle branch block. Chlamydia pneumoniae, Mycoplasma pneumoniae, herpes simplex virus, respiratory syncytial virus, adenovirus, and Coxsackie B virus antibody tests were negative. She was diagnosed with acute myocarditis. On the 11th day, her body temperature was 35.5°C and the arterial blood pressure was 81/59 mmHg. ECG monitoring showed bundle branch block and frequent premature and ventricular contractions. Blood tests showed elevated biomarkers of myocardial damage (peak creatin-kinase 1472 U/L, creatin-kinase MB 112 U/L, and lactate dehydrogenase 578 U/L). Fulminant myocarditis complicated by heart failure, arrhythmia, and a state of shock were considered. Supportive treatments with continued anti-infection medication were given. The right subclavian vein was catheterized, and arterial blood pressure and cardiac output were monitored. The patient suffered from sudden onset convulsions/seizure actives and received anti-seizure medication. ECG showed accelerated ventricular spontaneous rhythm and widened QRS, indicating severe myocardial damage. Color Doppler ultrasound showed poor cardiac function and abnormal ventricular wall motion, which further supported the diagnosis of fulminant myocarditis. The patient underwent right femoral vein puncture due to heart failure and renal insufficiency to perform bedside hemofiltration. On the 12th day, bedside ultrasound showed that the overall systolic function of the heart was reduced. The patient developed lactose acidosis and her blood pressure dropped. Blood tests showed raised biomarkers of myocardial damage (peak creatin-kinase 1727 U/L, creatin-kinase MB 223 U/L, and lactate dehydrogenase 3261 U/L). The patient developed ventricular fibrillation, bilateral mydriasis, loss of light reflex, persistently low blood pressure, and received chest compressions and extracorporeal membrane oxygenation (ECMO) therapy. Her condition deteriorated rapidly despite intensive medical treatments and supportive care. The patient died 12 days after COVID-19 vaccination.

A forensic autopsy was requested by her family because of the temporal relationship between COVID-19 vaccination and her death. The post-mortem study revealed light yellow effusion in the bilateral pleural cavities, 400 ml in the left, and 350 ml in the right. There was no thrombus in the pulmonary arteries. There was 70 ml of light-yellow effusion in the pericardial sac. The heart weighed 338.1 g, and the left anterior descending coronary artery and the right coronary artery had mild atherosclerosis. The spleen capsule was slightly shrunken, and the cut surface was pale. There was focal peripancreatic tissue hemorrhage, but no obvious hemorrhage was seen on the cut surface. No special structural finding was observed in the other organs.

**FIGURE 1:**
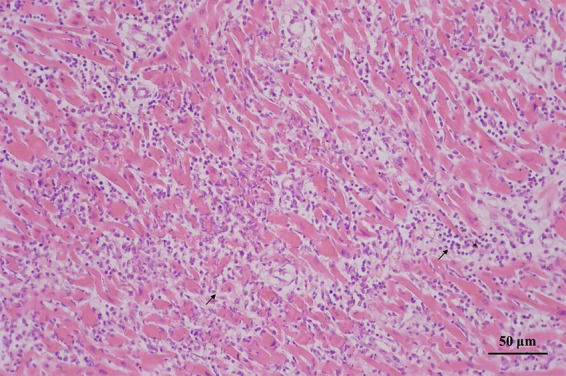
Myocardial degeneration and necrosis; myocardial interstitium is mainly infiltrated by lymphocytes and monocytes (arrows).

**FIGURE 2:**
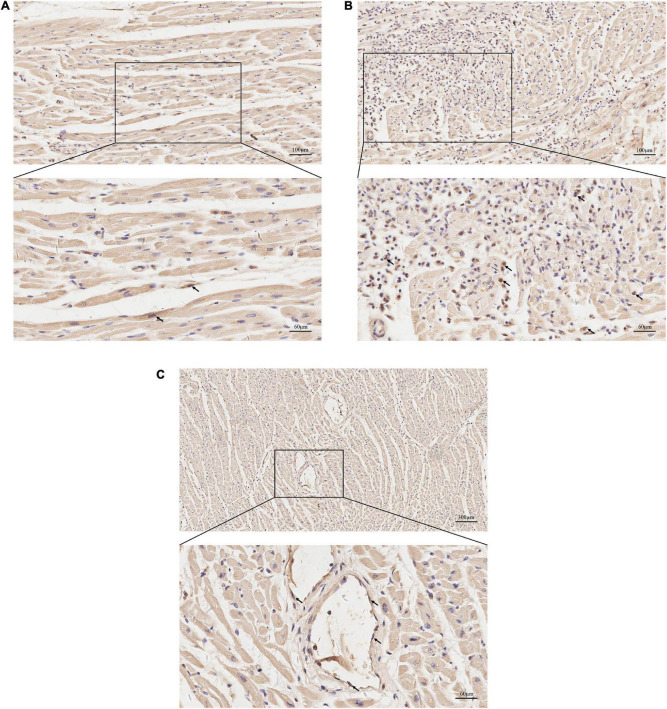
Immunohistochemical staining for protein expression of spike receptor binding domain (RBD) in heart sections. **(A)** Representative images of immunohistochemical staining of heart section showed occasional spike RBD positive cardiomyocytes (arrows). **(B)** Representative images showing spike RBD expression in the infiltrating immune cells in myocardium (arrows). **(C)** Representative images showing spike RBD expression in the vascular endothelial cells in myocardium (arrows).

Histological examination revealed that the left anterior descending coronary artery and the right coronary artery had mild atherosclerotic stenosis; the heart showed multi-focal inflammatory infiltration, mainly lymphocytes and monocytes associated myocyte degeneration and necrosis of both left and right ventricular walls (Figure 1). Histological examination of the tissues confirmed myocarditis. Immunohistochemical staining of heart section showed occasional spike RBD positive cardiomyocytes, infiltrating immune cells and vascular endothelial cells (Figures 2A–C). The lungs showed edema with local inflammatory cell infiltration (Supplementary Figure 1). Intracerebral vascular filling was poor, and the perivascular space was enlarged (Supplementary Figure 2A). The liver showed steatosis. The liver showed autolysis (Supplementary Figure 2B). In addition, Toluidine blue staining of the larynx, epiglottis, gastrointestinal, myocardium, lungs, and other tissues showed no mast cell infiltration or degranulation (Supplementary Figure 3).

## Discussion

On July 9, 2021, the WHO Global Advisory Committee on Vaccine Safety (GACVS) COVID-19 subcommittee published a report on myocarditis and pericarditis following COVID-19 mRNA vaccination, usually after the second dose of mRNA COVID-19 vaccine (7). Data showed that the estimated incidence of cardiac inflammation per 100,000 doses of CoronaVac (inactivated virus vaccine) and BNT162b2 (mRNA vaccine) was 0.31 (95% CI, 0.13–0.66) and 0.57 (CI, 0.36–0.90), respectively (8). Literature also reported that the risk of myocarditis and pericarditis increased after vaccination with an mRNA COVID-19 vaccine, and some scholars have proposed that the use of viral vector vaccine alternatives may reduce the occurrence of myocardial damage after vaccination (9).

Current studies suggest that the possible pathogenesis of mRNA COVID-19 vaccine-induced myocarditis is mainly molecular mimicry and delayed allergic reaction (10). An important underlying mechanism of myocarditis after mRNA COVID-19 vaccination is the molecular mimicry between the spike protein of the novel severe acute respiratory syndrome coronavirus (SARS-CoV-2) and self-antigens (11). The surface spike protein of SARS-CoV-2 serves as an mRNA vaccine-encoded immunogenic target that may induce immune cross-reactivity through molecular mimicry of live virus infection (12). Elevated levels of cardioreactive autoantibodies have been reported in patients with myocarditis following COVID-19 vaccination (13). These antibodies may target multiple antigens, and when cells cannot distinguish between foreign and self-peptides, pro-inflammatory cytokine responses are dysregulated, resulting in immune damage to the host. Another possible hypothesis is delayed hypersensitivity. Phagocytic and eosinophilic infiltration and mild peripheral eosinophilia were found in cardiac tissue of patients following COVID-19 vaccination (4). In fact, the patients may have been sensitized by the first dose of the vaccine and may have then experienced delayed hypersensitivity reactions following the second dose. This would explain why most patients did not develop symptoms of myocarditis until 2–4 days after the second dose of the vaccine.

Most reported patients with myocarditis following COVID-19 vaccination were vaccinated with mRNA vaccines, and myocarditis caused by other types of COVID-19 vaccines was rarely reported. A case-control study of the risk of carditis associated with two different types of COVID-19 vaccines found an increased risk of myocarditis after mRNA COVID-19 vaccination, but no association between inactivated virus vaccines and myocarditis was reported in the literature (8). The patient reported in this article was vaccinated with the protein subunit COVID-19 vaccine (ZF2001). ZF2001, produced by Anhui Zhifei Longcom Biologic Pharmacy Co., Ltd., is an RBD-dimer subunit vaccine with conditional approval in China. SARS-CoV-2 RBD antigen was manufactured in the CHO cell lines as a liquid formulation containing 25 μg per 0.5 mL, and aluminum hydroxide was used as an adjuvant for the antigen (14, 15). To our knowledge, this is the first reported case of myocarditis following this type of COVID-19 vaccine.

Myocarditis can be one of the clinical symptoms in patients with COVID-19 infection (16). In addition, there are many other viruses can cause myocarditis. Viral myocarditis may be followed by bacterial and fungal infections. Non-infectious factors such as drugs and allergic reactions can also lead to myocarditis (17). Therefore, SARS-CoV-2 and common myocarditis-causing viruses, such as Coxsackie group B virus, parvovirus B19, and herpes virus, should be tested in patients with suspected post- COVID-19 vaccinated myocarditis to rule out SARS-CoV-2 and viral infections. In this case, the new coronavirus *ORF1a* gene test was negative, and the new coronavirus *N* gene test was negative, indicating that the patient did not have COVID-19 infection. In addition, the antibody tests for respiratory syncytial virus, adenovirus, Coxsackie B virus, herpes simplex virus, and hepatitis virus were also negative. Clinical presentation showed that there was a clear temporal relationship between the protein subunit of COVID-19 vaccine and the occurrence of myocarditis in this patient. Although potential cause of myocarditis due to viral infection cannot be completely excluded because no viral culture/viral DNA/RNA detection were performed, myocarditis could be a severe side effect of the protein subunit of COVID-19 vaccine.

## Conclusion

We reported a case of fulminant myocarditis following the first dose of the ZF2001 RBD-subunit vaccine against COVID-19. There has been increasing data supporting the association between vaccination and myocarditis, but the exact mechanism by which different types of COVID-19 vaccines mediate the development of myocarditis is still unclear. Further research is needed to address the specific mechanism of myocarditis after COVID-19 vaccination and to discover specific biomarker that can indicate myocarditis or myocardial damage after COVID-19 vaccination.

## Data availability statement

The original contributions presented in this study are included in the article/Supplementary material, further inquiries can be directed to the corresponding authors.

## Ethics statement

The studies involving human participants were reviewed and approved by the Medical Ethics Committee of Hebei Medical University. The patients/participants provided their written informed consent to participate in this study.

## Author contributions

Y-MD wrote the manuscript, while G-ZZ and BC critically revised it. Y-MD, XL, C-TY, QQ, W-BS, Y-ML, MZ, S-JW, H-TB, R-FM, G-ZZ, and BC collected the data upon which the manuscript was based, discussed the results, and reviewed and approved the manuscript. All authors contributed to the report and approved the submitted version.
