# Supplementary material for: Case report: Myocarditis following COVID-19 protein subunit vaccination
Source: Front Cardiovasc Med. 2022 Sep 7;9:970045. doi: 10.3389/fcvm.2022.970045 (PMC9489902; doi:10.3389/fcvm.2022.970045)
Supplement: Supplementary file 1 [file Data_Sheet_1.docx]

Supplementary Material

# Supplementary Figures

## Supplementary Figure 1


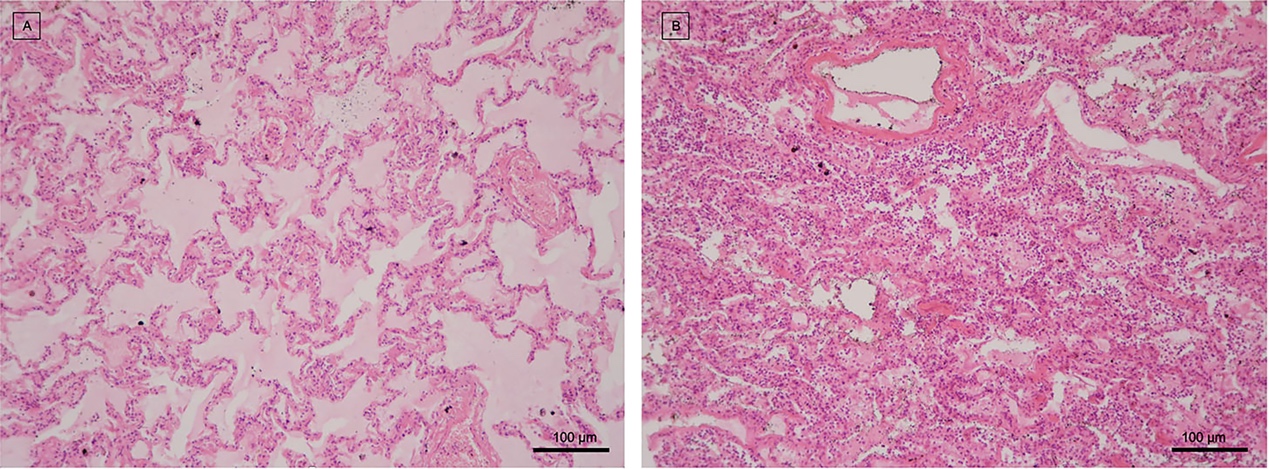


Supplementary Figure 1: HE stained images of lung tissue. A, Pulmonary edema. B, Local inflammatory cell infiltration in the lungs.

## Supplementary Figure 2


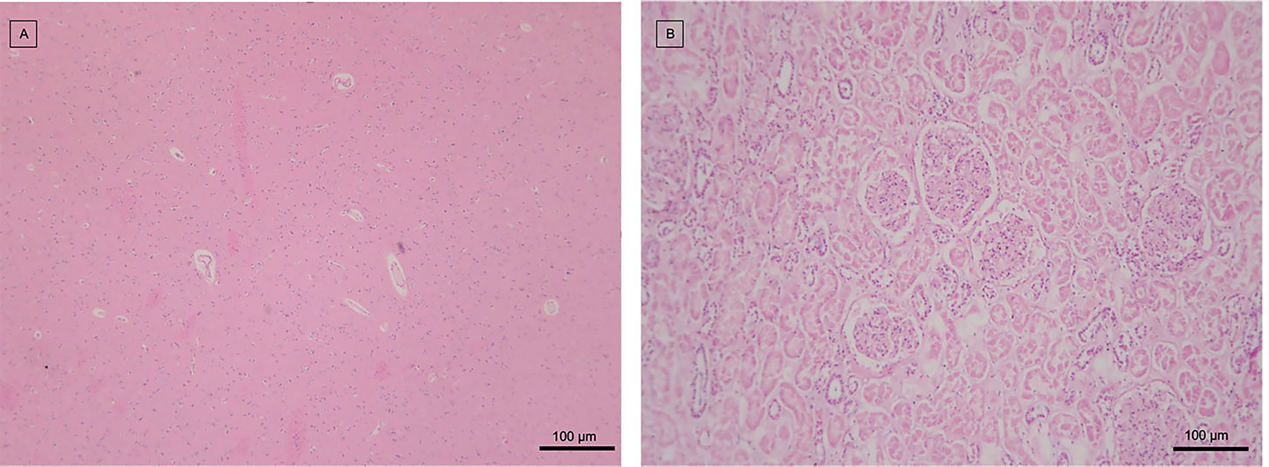


Supplementary Figure 2: HE stained images of brain and kidney. A, Poor vascular filling in the brain and increased space around. B, Autolysis in kidneys.

## Supplementary Figure 3


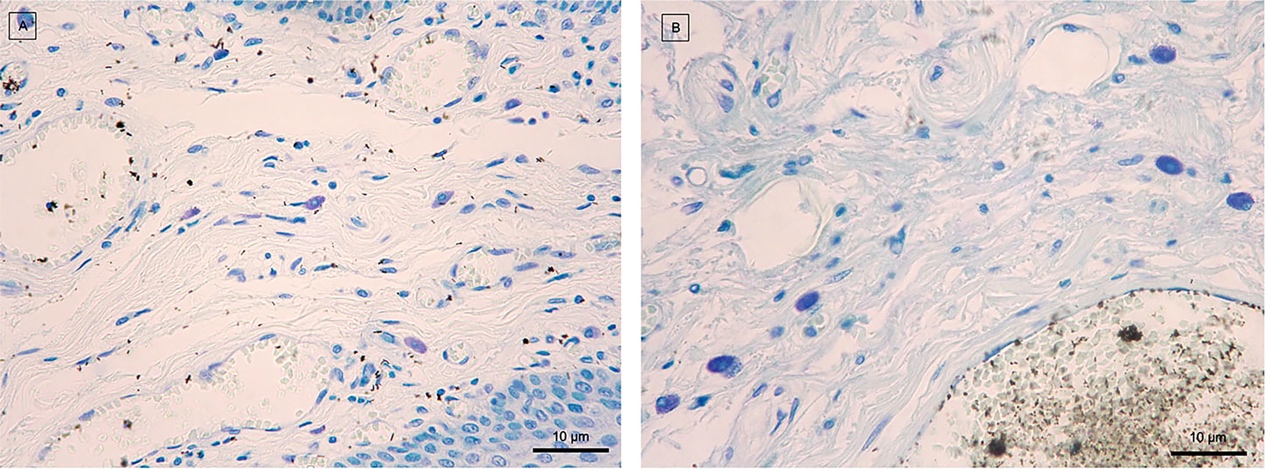


Supplementary Figure 3: Toluidine blue staining images. A-B, Toluidine blue staining of the larynx and epiglottis showed no mast cell degranulation.
